# The allylic chalcogen effect in olefin metathesis

**DOI:** 10.3762/bjoc.6.140

**Published:** 2010-12-23

**Authors:** Yuya A Lin, Benjamin G Davis

**Affiliations:** 1 Department of Chemistry, University of Oxford, Chemistry Research Laboratory, 12 Mansfield Road, Oxford OX1 3TA, United Kingdom

**Keywords:** allyl substituent effect, allyl sulfides, aqueous chemistry, olefin metathesis, protein modifications

## Abstract

Olefin metathesis has emerged as a powerful tool in organic synthesis. The activating effect of an allylic hydroxy group in metathesis has been known for more than 10 years, and many organic chemists have taken advantage of this positive influence for efficient synthesis of natural products. Recently, the discovery of the rate enhancement by allyl sulfides in aqueous cross-metathesis has allowed the first examples of such a reaction on proteins. This led to a new benchmark in substrate complexity for cross-metathesis and expanded the potential of olefin metathesis for other applications in chemical biology. The enhanced reactivity of allyl sulfide, along with earlier reports of a similar effect by allylic hydroxy groups, suggests that allyl chalcogens generally play an important role in modulating the rate of olefin metathesis. In this review, we discuss the effect of allylic chalcogens in olefin metathesis and highlight its most recent applications in synthetic chemistry and protein modifications.

## Review

**Scheme 1:**
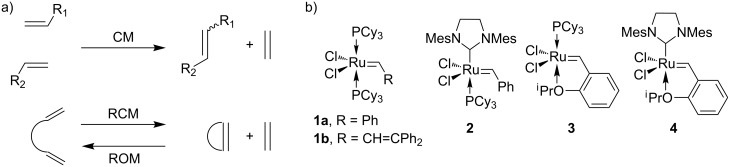
a) Variation of olefin metathesis: CM = cross-metathesis; RCM = ring-closing metathesis; ROM = ring-opening metathesis. b) Conventional ruthenium-based metathesis catalysts.

Olefin metathesis is one of the most useful chemical transformations for forming carbon–carbon bonds in organic synthesis (Scheme 1) [1–4]. The broad utility of olefin metathesis is largely due to the exquisite selectivity and the high functional group compatibility of ruthenium-based metathesis catalysts. Catalysts such as **1**–**4** were found to tolerate many functional groups also found in biomolecules, including amides, alcohols, and carboxylic acids. In some cases, metathesis is even compatible with amine and sulfur containing building blocks. Together with its high stability in various media and excellent chemoselectivity, olefin metathesis has been used on peptide substrates for various applications in chemical biology [5–8]. The development of water-soluble metathesis catalysts [9–13] and other advances in aqueous metathesis such as the use of organic co-solvents [14–15], reviewed in detail recently [16], has enabled more recent examples of the reaction on protein substrates [17–18].

The outcome of olefin metathesis is sensitive to multiple factors, such as the nature of the catalyst, steric crowding around the alkene and the directing effects of nearby heteroatoms. These factors are of great importance, especially when optimizing reaction conditions for delicate natural product synthesis or protein modification. Interestingly, the activating effect of allylic heteroatoms, such as oxygen and sulfur in olefin substrates, has been observed in many examples and was found to play an important role for effective olefin metathesis in synthesis. These reports suggest that allylic chalcogens can modulate the rate of olefin metathesis, and their effects appear to be a general phenomenon in metathesis chemistry.

In this review, we collect these reports and discuss the activating effect of allylic chalcogens, such as oxygen, sulfur and selenium, as well as their relative reactivity in olefin metathesis. The applications of the allylic chalcogen effect in protein modifications via olefin metathesis and the associated principles of cross-metathesis (CM) partner selection for reliable and efficient reaction on proteins are also highlighted.

### The effect of allylic hydroxy groups in olefin metathesis

**Figure 1:**
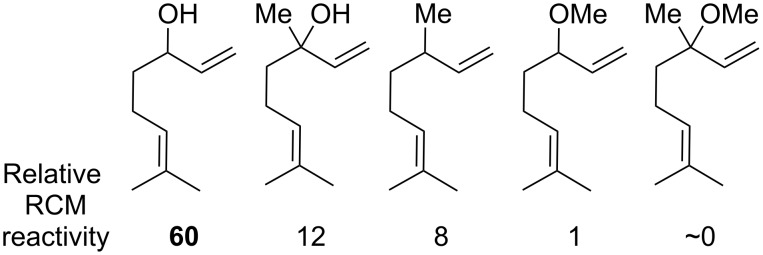
Allylic hydroxy activation in RCM [19].

The activating effect of allylic hydroxy groups in ring-closing metathesis (RCM) was first identified by Hoye and Zhao in 1999 [19]. In this work, the influence of both the steric and electronic character of allylic substituent of linalool and related analogues in RCM was assessed. The free hydroxy group on linalool greatly enhanced RCM relative to the corresponding methyl ether or unsubstituted analogues (Figure 1). This activating effect was marked and initially surprising given that *tert*-butylethylene, containing a fully substituted allylic center, was reported to be almost inert to reaction with catalyst **1** [20].

**Figure 2:**
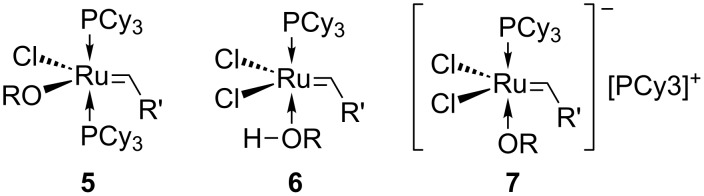
Possible complexes generated through preassociation of allylic alcohol with ruthenium.

A number of possible explanations for the rate acceleration due to allylic hydroxy groups in olefin metathesis have been proposed [19]. For example: The preassociation of the alcohol takes place at the ruthenium center through the rapid and reversible exchange of the alkoxy a for chloride ligand to give a complex such as **5**, or the exchange of the alcohol for a phosphine ligand exchange to generate **6**. The anionic complex **7** could also promote the reaction (Figure 2). Hydrogen bonding between the allylic hydroxy group on the substrate and the chloride ligand on the catalyst could also be another reason that favors subsequent metathesis events. Since the formation of **5** is unlikely under the reaction conditions reported by Hoye and Zhao [19], and species **6** and **7** would prevent metathesis proceeding further, the most plausible explanation for the positive effect of allylic hydroxy in olefin metathesis, bearing in mind that allyl ethers do not show such an effect, is through hydrogen-bonding to the catalyst.

Following Hoye and Zhao’s observations, several organic chemists have further studied the effect of allylic alcohols and ethers in metathesis, and some have taken advantage of this activating effect for efficient and selective construction of complex molecules over the past 10 years. Here we outline a few pertinent and illustrative examples.

**Scheme 2:**
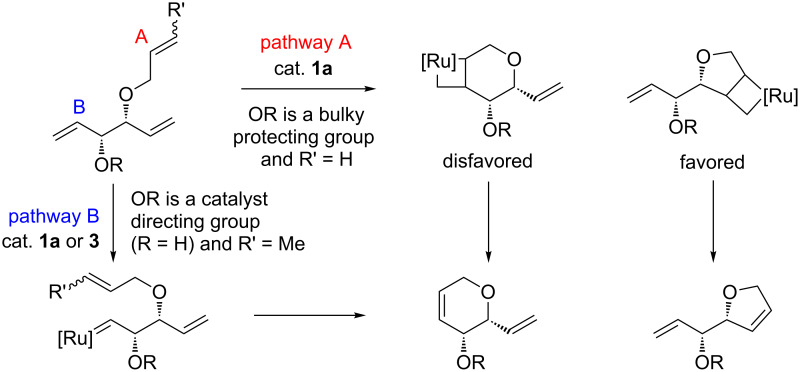
The influence of different OR groups on ring size-selectivity [21].

Schmidt prepared enantiomerically pure dihydropyrans and dihydrofurans bearing an unsaturated olefin tether based on a ring size-selectivity RCM reaction of a triene [21]. In this study, it was found that trienes containing a bulky hydroxy protecting group at the allylic position cyclized selectively to dihydrofurans, whereas the free alcohol yielded 6-membered rings with very high selection (Scheme 2).

This useful selectivity was attributed to the directing effect of the allylic hydroxy group. When the allylic alcohol was protected with a bulky group, the catalyst reacted preferentially at the less hindered allyl ether (pathway A where R′ = H). The formation of the 4,6-bicyclic intermediate is apparently disfavored with catalyst **1a** leading to largely the formation of the dihydrofuran product. It should be noted that when a more reactive second-generation catalyst (**2**) is used the selectivity for the dihydrofuran product is reduced. This selectivity was also found to be highly dependent on the size of protecting group. Significant decrease in ring-size selectivity was observed when smaller protecting groups, such as a methyl group, were used. Furthermore, when no protecting group was used (i.e., R = R′ = H, Scheme 2) the RCM reaction was unselective and resulted in a 1:1 mixture of 5- and 6-membered ring products. The selectivity for dihydropyran formation was therefore tuned by substitution at the terminal position of the allyl ether (e. g., R′ = Me), which directed the catalyst to react via pathway B. The authors were able to obtain significantly improved conversions and yields while maintaining high selectivity for dihydropyran by using, instead of **1a**, the Hoveyda–Grubbs first generation catalyst (**3**), where the catalytically active species is stabilized by a hemilabile benzylidene ligand. The activating effect of the allylic hydroxy group in RCM is further supported by the dramatic decrease in conversion when the free OH group was protected (i.e., R ≠ H, R′ = Me) in pathway B.

**Scheme 3:**
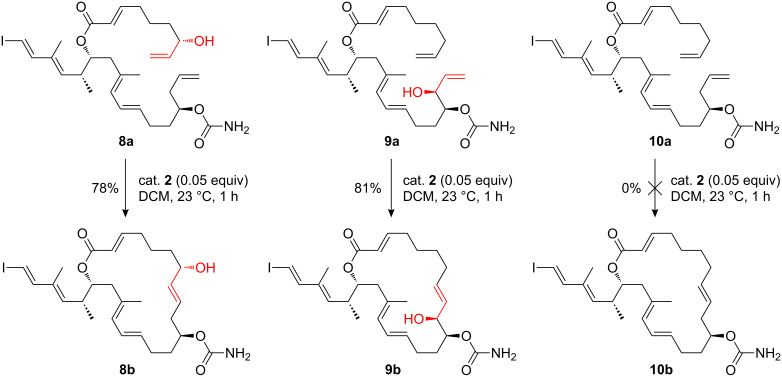
Synthesis of palmerolide A precursors by Nicolaou et al. illustrates enhancement by an allylic hydroxy group in an RCM strategy [22].

Pertinent examples have also emerged during target syntheses. In the synthesis of palmerolide A analogues by Nicolaou and co-workers, compounds **8a** and **9a** were found to undergo smooth macrocyclization via RCM, whereas **10a**, lacking the allylic hydroxy group, failed to form the desired macrocycle under the same conditions (Scheme 3) [22]. When **10a** was treated under more stringent conditions, decomposition and/or polymerization occurred. These observations suggest that the presence of an allylic hydroxy group in the molecule was crucial for enhancing the reactivity under the mild RCM conditions required by the potentially labile natural product scaffold.

**Scheme 4:**
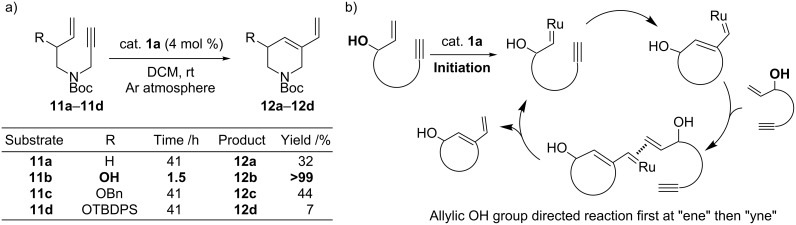
a) Acceleration of ring-closing enyne metathesis by the allylic hydroxy group [23]. b) Proposed mode of action by the allylic hydroxy group in this reaction.

Enhancement effects by an allylic hydroxy group have also been found in ring-closing enyne metathesis. Studies by Takahata et al. revealed that the ring-closing enyne metathesis of terminal alkynes containing an allylic hydroxy group proceeded smoothly without the ethylene atmosphere that is generally required to drive such reactions (Scheme 4a) [23]. Compound **11b** containing the allylic hydroxy group cyclized to the desired diene product **12b** with quantitative yield in 1.5 hours, whereas **11a**, without the allylic hydroxy group, required 41 hours to afford **12a** with a yield of only 32%. With the substituted allyl ethers **11c** and **11d**, reduction in yield was observed with increasing bulk of the protecting group (44% and 7%, respectively). Taking advantage of the allylic hydroxyl substituent effect, the authors synthesized (+)-isofagomine with the efficient ring-closing enyne metathesis of the acyclic starting material as the key cyclization step. Associated mechanistic studies suggested that the reaction proceeded via an “ene-then-yne” pathway, further suggesting that rate acceleration is likely due to the directing effect of the allylic hydroxy group on substrates (Scheme 4b).

**Scheme 5:**
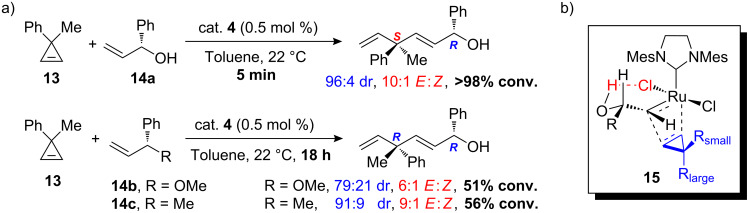
a) Effect of the hydroxy group on the rate and steroselectivity of ROCM [24]. b) Proposed H-bonded ruthenium complex for stereoselective ROCM.

As discussed earlier, the most likely explanation for the observed rate accelerations by the allylic hydroxy groups is hydrogen-bonding. Hoveyda and co-workers recently utilized hydrogen-bonding between the allylic hydroxy group and the ruthenium catalyst for stereoselective ring-opening cross-metathesis (ROCM) (Scheme 5) [24]. The activating effect from the allylic hydroxy group in metathesis is prominent in this example. The ROCM of cyclopropene **13** with enantiomerically enriched allylic alcohol **14a** is complete in 5 minutes (>98% conversion) with a high diastereomeric ratio (dr) (96:4) and *E*:*Z* selectivity (10:1) favoring the *S*,*R*-diasteromer. In contrast, the reaction of methyl ether **14b** and the methyl analogue **14c** is far less effective (51% and 56% conversion, respectively, in 18 hours) with lower and opposite stereoselectivity in favor of the *R*,*R*-diastereomers (Scheme 5a). The observed stereoselectivity can be explained by intramolecular hydrogen-bonding between the hydroxy group and the chloride ligand that results in a favored alkylidene intermediate complex with the substituted group of the stereogenic center situated away from the bulky mesityl groups. On coordination of the cyclopropene to the catalyst, the formation of a metallacyclobutane such as **15** with the larger R group pointing away from the main bulk of the catalyst is preferred (Scheme 5b). This intermediate then collapses to give the product with the observed stereoselectivity.

**Scheme 6:**
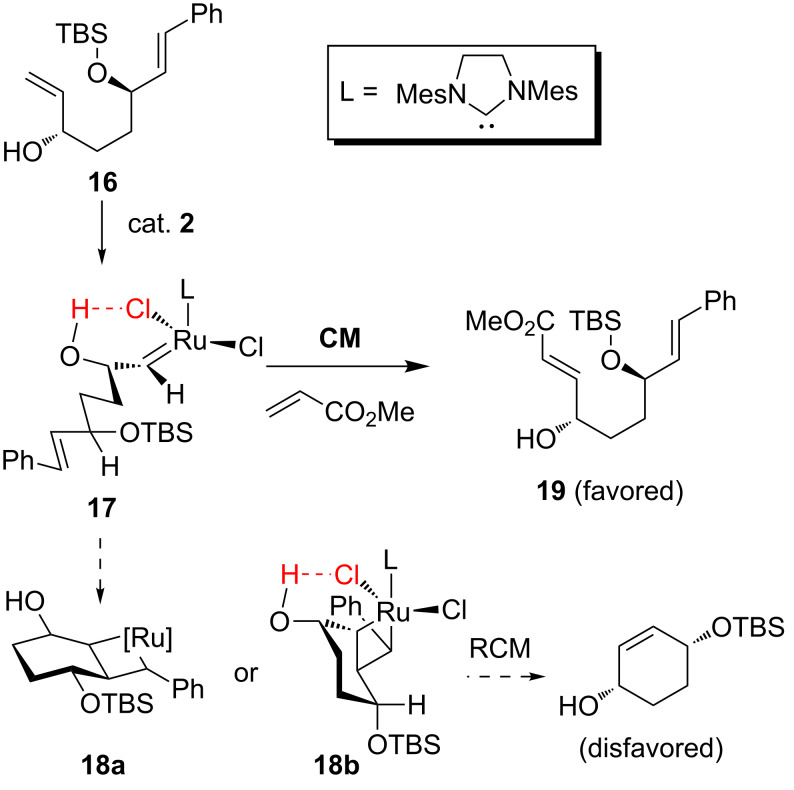
Plausible explanation for chemoselective CM of diene **16** [25].

Sasaki and co-workers have also exploited both allylic and homoallylic hydroxy as directing groups in olefin metathesis for selective formation of key fragments in two recent examples of natural product syntheses. In the synthesis of aspergillide A, a key fragment **19** was synthesized by the CM reaction between diene **16** and methyl acrylate with very high yield [25]. Remarkably, none of the possible RCM products of the diene were detected. This observed chemoselectivity was ascribed to hydrogen-bonding of the allylic hydroxy to the chloride ligand of the catalyst resulting in an intermediate alkylidene **17** which has a conformation unfavorable for RCM. The open chain intermediate **17** favors CM, while RCM of **16**, must proceed via unfavorable higher energy conformations such as **18a**, generated by breaking hydrogen-bonding in **17**, and/or highly strained intermediate **18b**, if the reaction was to occur (Scheme 6). Similar directing effects of homoallylic alcohol in olefin metathesis have also been utilized in the concise synthesis of (+)-neopeltolide by the same group [26].

It should be noted that all of the illustrated examples regarding allyl hydroxy activation in olefin metathesis are predominantly secondary allyl alcohol substrates. The lack of primary allyl alcohol examples can be explained by the fact that, in general, these dehydrogenate at elevated temperature in the presence of ruthenium based metathesis catalysts. These in turn form ruthenium hydride species, which are effective catalysts for isomerization of alkene substrates [27–28].

### The effect of other allylic chalcogens in olefin metathesis

#### Allyl sulfides are privileged substrates in olefin metathesis

**Scheme 7:**
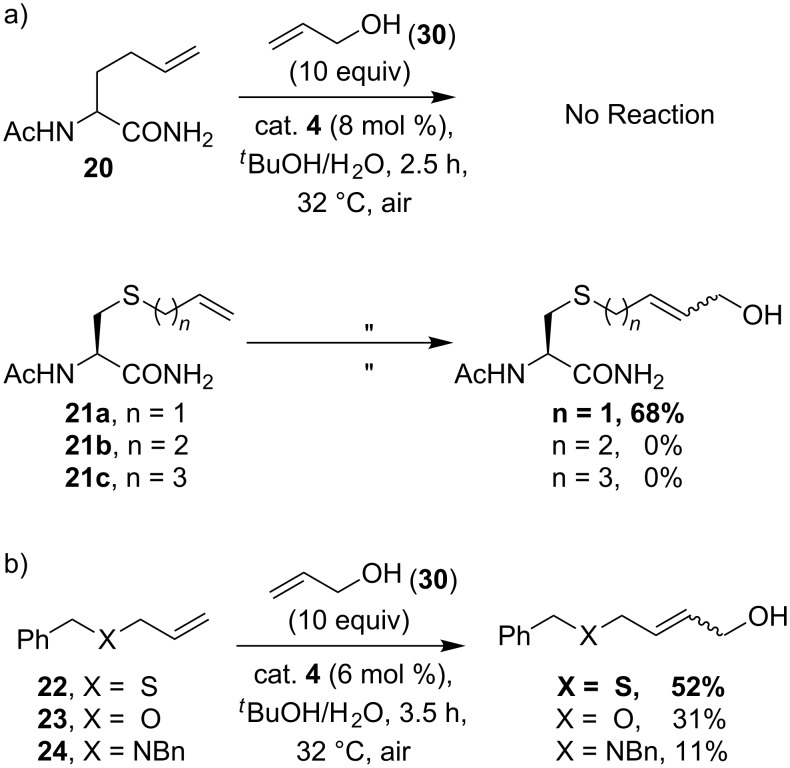
a) Efficient cross-metathesis of *S*-allylcysteine [17]. b) Comparison of relative reactivity between allylic heteroatom derivatives.

While there are many examples of allylic alcohols and ethers in metathesis, examples with allyl sulfide substrates were until recently noticeably few. This is unsurprising since sulfur-containing molecules are often detrimental in many transition-metal-catalyzed transformations. Indeed, there have been several cases of olefin metatheses in which sulfides were problematic [29–32]. In our exploratory work in aqueous metathesis, cross-metathesis of unsaturated amino acids with allyl alcohol mediated by catalyst **4** was investigated. Unexpectedly, *S*-allylcysteine derivative **21a** was the only substrate that afforded a synthetically useful amount of CM product, whereas the reaction of the all carbon analogue homoallylglycine (**20**) and sulfide derivatives, *S*-butenyl and *S*-pentenyl cysteine (**21b** and **21c**, respectively), failed to work under identical conditions in aqueous media (Scheme 7a) [17]. In order to compare the relative CM reactivity between other allylic heteroatom derivatives, further studies were carried out on the CM of allyl benzyl ether (**23**) and allyl dibenzylamine (**24**), but the allyl sulfide analogue **22** remained the most reactive substrate in aqueous media (Scheme 7b) [17].

**Scheme 8:**
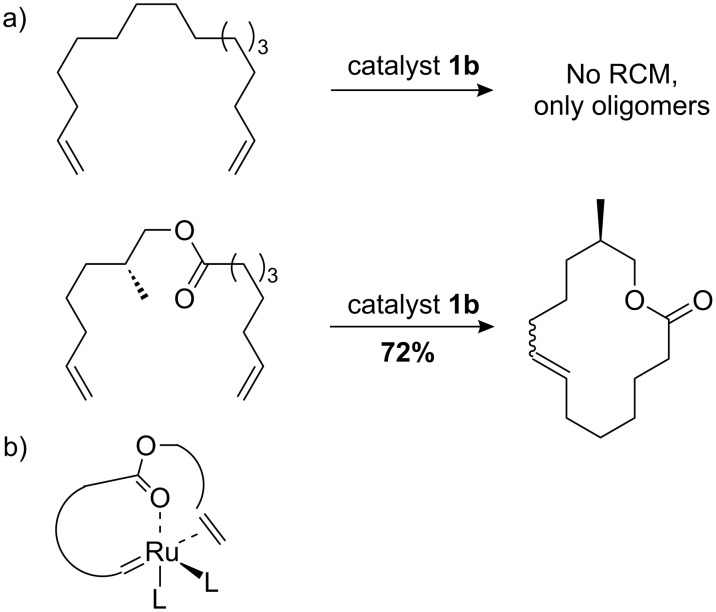
a) Macrocycle synthesis by carbonyl-relayed RCM. b) Putative complex in carbonyl-relayed RCM [33].

**Scheme 9:**
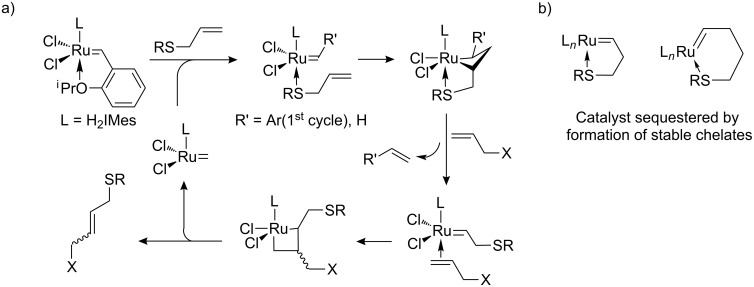
a) Sulfur assisted cross-metathesis [17]. b) Putative unproductive chelates for larger ring sizes generated from butenyl or pentenyl sulfides.

We considered these observation in the light of the early work by Fürstner in the synthesis of macrocycles by RCM [33], in which a “carbonyl-relayed” mechanism was proposed as the explanation for favorable macrocyclization by RCM over oligomerization (Scheme 8a). Here, the coordination by the carbonyl oxygen to ruthenium brings the tethered alkene closer in proximity to the alkylidene allowing effective cyclization (Scheme 8b). In a similar manner, the rate enhancement caused by allyl sulfide could be explained with a sulfur relayed mechanism (Scheme 9a), where sulfur pre-coordination to the ruthenium center increases the effective concentration between the alkylidene and the alkene substrate. For the allyl sulfides this can occur without detrimental chelation, which is thought to be the case for butenyl and pentenyl sulfides (Scheme 9b). Grela and Lemcoff have synthesized the thio-derivatives of the Hoveyda-type catalyst and found that they initiate at much higher reaction temperature [34]. Their finding is in agreement with our observations of butenyl sulfides **21b**, which resulted in no productive CM at room temperature. This is presumably due to the formation of 5-membered stable chelates such as those depicted in Scheme 9b.

In the few examples where allyl sulfides had previously been used in olefin metathesis [32,35–36], the prior focus of attention had been the tolerance of the catalyst for sulfur; the enhanced reactivity relative to other alkenes was apparently unnoticed. Our results showed that allyl sulfides are not simply tolerated as they can *enhance* the rate of olefin metathesis in a similar yet more effective way compared to allyl alcohols and ethers. This is likely due to the soft nature of sulfur as a Lewis base making it a better ligand for ruthenium than the oxygen atom in Hoveyda–Grubbs second-generation catalyst **4**. It is currently unclear whether a similar effect would be observed for phosphine-containing metathesis catalysts such as **1a** and **2**. As Hoye and others have realized, the presence of an allyl alcohol can also potentially accelerate catalyst decomposition [19,27–28]. The use of allyl sulfides in these systems is not exceptional, especially when aqueous solvents are used. However, the reaction with allyl sulfides proved to be sufficiently high in turnover frequency that it outcompetes catalyst decomposition, a likely key aspect of its success in water.

**Scheme 10:**
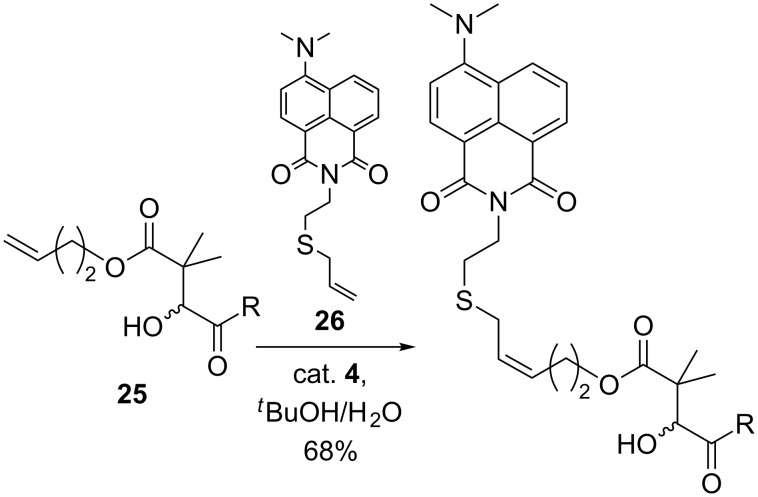
Functionalization of Mukaiyama aldol product by CM in aqueous media [37].

In recent work by Loh and co-workers, an allyl sulfide derivative was utilized in synthesis precisely for its enhanced metathesis reactivity [37]. Adopting the reaction conditions previously optimized by our group, compound **25** was efficiently functionalized with an allyl sulfide derived fluorescent probe **26** via CM in aqueous media to demonstrate the utility of functionalization of peptides and proteins by the Mukaiyama aldol reaction (Scheme 10).

#### Allyl selenides are superior metathesis substrates to allyl sulfides in aqueous cross-metathesis

**Scheme 11:**
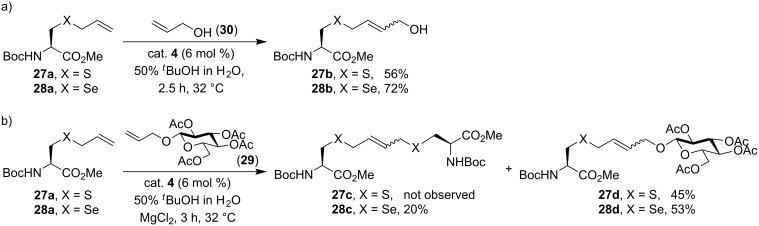
Comparison of reactivity between allyl sulfides and allyl selenides in aqueous cross-metathesis [38].

Based on our results on allyl sulfides, alongside reports on the activating effect of allylic alcohols and ethers, the positive influence of allyl chalcogens on the rate of olefin metathesis is obvious. We naturally extended our investigation to allyl selenides, the next element in the group, expecting it to have a similar influence as oxygen and sulfur in metathesis. *Se*-allylselenocysteine derivative **28a** was tested along with the allyl sulfide analogue **27a** in model aqueous CM with allyl alcohol under identical reaction conditions. The allyl selenide **28a** was found to be more effective than the allyl sulfide case in CM, with respective yields of 72% and 56% (Scheme 11a). The CM reaction with a more complex and biochemically relevant carbohydrate substrate **29** was also examined. Indeed, the allyl selenide was overall more reactive than allyl sulfide with combined CM yields of 73% (CM and self-metathesis) and 45% (CM only, no self-metathesis observed), respectively (Scheme 11b) [38].

This further improvement in reactivity may be attributed to the increased softness of selenium which makes the coordination to ruthenium even more favorable than the sulfur in allyl sulfides. While, as a single example, Kotetsu and co-workers have synthesized selenium-containing bicyclic β-lactams via RCM of an allyl selenide derivative, enhanced reactivity was unnoticed [39]. With a better understanding of the allylic chalcogen effect, olefin metathesis has been further exploited in protein modifications. This is discussed next.

### Applications in protein modifications

For the potential use of olefin metathesis in bioconjugation, the genetic incorporation of alkene containing amino acid residues has been well documented [18,40–44]. However, the reaction had been unsuccessful until the recent realization of the effect of allyl chalcogens, allyl sulfides especially, in enhancing the rate of aqueous metathesis [17].

**Scheme 12:**
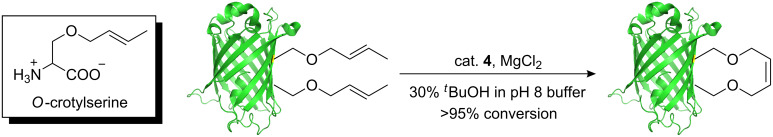
Ring-closing metathesis on a protein [18].

Ai et al. have recently reported an example of RCM on a protein with genetically encoded alkene residues [18]. *O*-Crotylserine containing substituted allyl ether was chosen as the residue for incorporation for two reasons: The beneficial effect of allyl heteroatoms in metathesis, and the more water-stable propagating catalytic species generated from the substituted alkene compared with the methylidene that results from terminal olefins [45]. Indeed, the RCM on the double *O*-crotylserine mutant proceeded with near-complete conversion after 5 hours (Scheme 12).

**Scheme 13:**
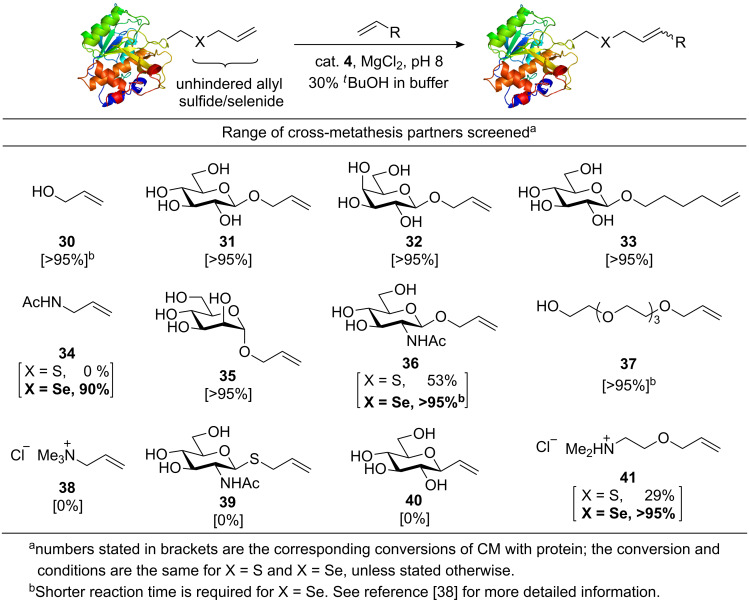
Expanded substrate scope of cross-metathesis on proteins [38].

Our group utilized the enhanced reactivity of allyl sulfides in aqueous cross-metathesis for application to protein modifications [17]. Very recently, with an aim to develop olefin metathesis as a more general method for bioconjugation, we have considered various key factors including steric, electronic and allyl linker selection in substrates that contribute to successful CM on proteins (Scheme 13) [38].

These studies suggest that an unhindered allyl sulfide or selenide protein tag is a requirement for most effective CM. Allyl and hexenyl ethers were found to be the most compatible CM partners for allyl sulfide or selenide containing proteins. The more challenging alkene substrates, such as the ones containing electron-deficient *N*-acetylamine, GlcNAc and ethanolamine (compounds **34**, **36** and **41**), required the more reactive allyl selenide protein tag for the CM to proceed efficiently. Reactive allyl sulfides such as **39** were unsuitable as CM partners for the protein substrate since they led predominantly to self-metathesis. The allyl amine derivative **38** was unreactive in metathesis with proteins possibly due to an electron-deficient or sterically-demanding nature. Taking these factors into consideration, effective functionalization of proteins by CM (>95% conversion) was achieved with 9 different substrates including biochemically important molecules such as GlcNAc, mannose and *N*-acetylamine, which could serve as effective mimics of post-translational protein modifications (glycosylation, lysine acetylation).

## Conclusion

Since the early work by Hoye on secondary allylic alcohols [19] and later the studies on allyl sulfides by our group [17], the allyl chalcogen effect has affected the way chemists use metathesis in synthesis and chemical biology. Complex molecules and metathesis partners can be joined efficiently with the aid of the natural affinity of ruthenium for allyl chalcogens. In this review, we have highlighted various applications of olefin metathesis in synthesis and protein modifications where the positive influence of allyl chalcogens is utilized. These reports, now collected here, suggest that the directing effect of allyl chalcogens is indeed a general phenomenon in metathesis chemistry, and allow a better understanding of the metathesis reaction itself. We hope to see this concept being further exploited in bioconjugation and synthetic chemistry.
